# Supplementary material for: Which social media platforms facilitate monitoring the opioid crisis?
Source: PLOS Digit Health. 2025 Apr 28;4(4):e0000842. doi: 10.1371/journal.pdig.0000842 (PMC12036940; doi:10.1371/journal.pdig.0000842)
Supplement: S1 Text — Includes details of platform shortlisting methods, term lists, algospeak generation protocol, per-platform content restriction policies and data access policies, and equations. (DOCX) [file pdig.0000842.s001.docx]

### Supplementary Text

Table of Contents

- Appendix A. Details of platform shortlisting methods
- Appendix B. Formal opioid term list
- Appendix C. Informal opioid term list
- Appendix D. Algospeak opioid term list
- Appendix E. Algospeak generation protocol
- Appendix F. Common noun / “household term” list
- Appendix G. Current content restriction policies, by platform (as of February 1, 2024)
- Appendix H. Current data access policies, by platform (as of December 1, 2023)
- Appendix I. Supplementary references
- Equation A
- Equation B
- Equation C

**Appendix A.** **Details of platform shortlisting methods**

We considered a platform to be active if we were able to access a website on an internet browser or if a mobile application was available for download. We chose to restrict our platform list to include only those that have an active website or mobile application to identify opportunities for future prospective research.

We classified platforms as social media following a definition used by the Knight First Amendment Institute at Columbia University (Rajendra-Nicolucci *et al.* 2021): social media is defined as “a digital space that combines communicating or sharing media with aspects of social networking sites,” and only includes platforms that are primarily social in nature (e.g. excluding news sites with comment sections, marketplaces, and content subscription services).

We considered a platform to be primarily for private messaging if its only features were person-to-person or group messages. We excluded these platforms as their data is not accessible for research use.

We only included platforms that were based in the United States or Canada, or that used English as the default language for a user based in the United States. While opioid misuse and addiction is a global crisis, we chose to focus on North America given the severity of the opioid epidemic in the region (Giorgi *et al.* 2023; Matero *et al.* 2023).

To estimate how much opioid-related discussion exists on each platform, we used the Google Search API to query for a set of opioid-related keywords (**Appendix B**). We selected only keywords that are specific to opioid use. If the query returned more than 25,000 hits for a platform, we considered that platform to have a high volume of opioid-related discussion.

Two authors (ATN, IAS) independently assessed each platform. The authors compared their assessments, identified discrepancies in their judgements, and independently completed a second review of platforms for which there were disagreements. The authors then discussed their secondary evaluations until they agreed on the assessment of each platform.

**Appendix B. Formal opioid term list**

fentanyl, opioids, opiates, morphine, codeine, oxycodone, oxymorphone, mscontin, percocet (n = 9)

**Appendix C. Informal opioid term list**

sublimaze, duragesic, fentanil, sufentanil, fentanylum, fentora, thebaine, codiene, roxanol, kadian, oxycontin, roxicodone, roxicet, endocet, endocodone, oxyir, oxynorm, hydrocodone, vicodin (n=19)

**Appendix D. Algospeak opioid term list**

paink!ller, f3nt@nol, cod3in3, c0d0n3, f3nt4nol, p@1nk!ller, oxy80s, 0xy80, m0rph!n3, m3rph0n3, m0rf3n, c0d3in, 0xyc, s!zzurp, 0pana, 0xym0rph0ne, num0rph@n, m0rf33n (n = 18)

**Appendix E. Algospeak generation protocol**

We used the OpenAI Chat Completions API from Microsoft Azure to generate our algospeak slang terms. We used the gpt-4 model and the “2023-05-15” Azure API version. We set the temperature to 0.7.

We gave the model the following system message as context:

“You are an AI assistant that helps people find information. You are particularly hip with online slang and know everything about how people talk on social media platforms like Facebook, Twitter, Reddit, and TikTok.”

We used the following prompt template, which was developed through iterative prompt engineering in a sandbox environment:

“We are playing a game where I give you a word, and then you give me algospeak terms for that word. The algospeak must be in the format such that none of the included words would be present in an English dictionary. I will give you an example; please continue each of these comma-separated lists:

1. dog: d0g, pupper, doggo

2. die: d!e, unalive, d!3

3. [INSERT QUERY DRUG EXAMPLE]

4. friend: fr13nd, @mig0, frend

In your response, only give the four lists (for dog, die, [INSERT QUERY DRUG], and friend) with comma-separated terms. Do not include any other text.”

Using this template, we created prompts for each of the five opioids for which to generate algospeak terms. We replaced [INSERT QUERY DRUG] with one of codeine, fentanyl, morphine, oxycodone, or oxymorphone. We replaced [INSERT QUERY DRUG EXAMPLE] with the corresponding algospeak example list for the selected drug:

codeine: c0dein3, c0dine, cod eine

fentanyl: f3ntanyl, f3nt, f3ntanil

morphine: m0rphine, morf1ne, m0rph1n3

oxycodone: 0xy, 0xyc0d0n, p3rcs

oxymorphone: 0xym0rph0ne, 0pana, num0rph@n

All example algospeak terms were human-generated based on observed internet language patterns.

We acknowledge that this prompt setup, including the system context statement, could potentially find improvement with modification, particularly ensuring that all language reflects current common internet text patterns. However, because prompt engineering is a process that can iterate interminably if one is searching for an optimal prompt, and because we saw that this prompt produced desirable results, we have elected to use this prompt for a production run without undertaking additional optimization experiments.

We submitted each drug query to the Chat Completions API 100 times and processed the results to extract all generated algospeak drug terms. This yielded 300 codeine terms (74 unique), 303 fentanyl terms (167 unique), 310 morphine terms (169 unique), 310 oxycodone terms (143 unique), and 356 oxymorphone terms (198 unique). We eliminated any terms only generated once in an attempt to remove noise. This left 26 codeine terms, 32 fentanyl terms, 35 morphine terms, 47 oxycodone terms, and 41 oxymorphone terms. We also eliminated any terms that did not include numbers or special characters, as those without those characters did not fit our definition of “algospeak.” We eliminated any terms that had a corresponding “non-algospeak” term that did not have an opioid-specific meaning (*e.g.* the non-algospeak equivalent of “f3ntanil” is “fentanil”). To reduce the scale of the list to a similar scale as that of the formal and informal opioid terms, we kept only terms generated more than four times, which yielded a final list of 18 algospeak terms.

**Appendix F. Common noun / “household term” list**

time, way, life, place, friend, idea, name, death, son (n=9)

**Appendix G. Current content restriction policies, by platform (as of February 1, 2024)**

*Bluelight*

“Bluelight neither condemns nor condones the use of drugs. Rather, we accept that drug use will always exist irrespective of legal status or societal norms. While there is no truly safe way to use drugs, we understand that prohibition and abstinence are not realistic or desirable solutions for everyone, nor have they been adequate in addressing the serious public health concerns associated with drug use… These forums invite visitors to discuss addition and sobriety in a non-judgmental setting, share recovery resources and encourage members to seek help." (<https://bluelight.org/xf/pages/BL_AboutUs/>)

“[Y]ou may not use Bluelight in any way, shape or form for unlawful purposes, including, without limitation: attempting to solicit, obtain sell or supply contraband substances or substances of a quasi-legal status or requesting information on how to do so…” (<https://bluelight.org/xf/pages/BLUA/>)

*Drugs-forum*

“Requesting or offering illegal substances is not allowed. This includes chemicals on the UN red list, DEA List I or scheduled precursors as well as thresholds or above quantities of DEA List II chemicals. This will get you banned instantly… Requesting, offering or trading sources for illegal substances is not allowed. This will get you banned instantly… Do not advertise any commercial websites or products." (<https://drugs-forum.com/threads/the-rules.298373/>)

“As a result of our strict moderation, Drugs-Forum offers a safe space for discussion of all aspects of recovery and drug use, both medical and recreational.” (<https://drugs-forum.com/>)

*Facebook*

“Do not post content that: Attempts to buy, sell, trade, co-ordinate the trade of, donate, gift or ask for high-risk drugs. Admits to buying, trading or co-ordinating the trade of high-risk drugs by the poster of the content by themselves or through others. Admits to personal use without acknowledgment of or reference to recovery, treatment, or other assistance to combat usage. This content may not speak positively about, encourage use of, coordinate or provide instructions to make or use high-risk drugs. Coordinates or promotes (by which we mean speaks positively about, encourages the use of, or provides instructions to use or make) high-risk drugs. Do not post content that: Attempts to buy, sell, trade, co-ordinate the trade of, donate, gift or asks for non-medical drugs. Admits to buying, trading or co-ordinating the trade of non-medical drugs by the poster of the content by themselves or through others. Admits to personal use without acknowledgment of or reference to recovery, treatment, or other assistance to combat usage. This content may not speak positively about, encourage use of, coordinate or provide instructions to make or use non-medical drugs. Coordinates or promotes (by which we mean speaks positively about, encourages the use of, or provides instructions to use or make) non-medical drugs.” (<https://transparency.fb.com/policies/community-standards/restricted-goods-services/>)

*Instagram*

“[B]uying or selling non-medical or pharmaceutical drugs are also not allowed. We also remove content that attempts to trade, co-ordinate the trade of, donate, gift, or ask for non-medical drugs, as well as content that either admits to personal use (unless in the recovery context) or coordinates or promotes the use of non-medical drugs.” (<https://help.instagram.com/477434105621119>)

*LinkedIn*

“Do not promote, sell or attempt to purchase illegal or dangerous goods or services. We don’t allow content that facilitates the purchase of illegal or dangerous goods and/or services… We also don’t allow content depicting or promoting instructional weapon making, drug abuse, and threats of theft." (<https://www.linkedin.com/legal/professional-community-policies>)

*Pinterest*

“Pinterest isn’t a place for trading or selling of certain regulated goods—products or substances that can cause harm when used, altered or manufactured irresponsibly—or for the display or encouragement of dangerous activities. We limit the distribution of or remove such content and accounts, including: Individuals and unlicensed retailers offering to sell, purchase or trade alcohol, tobacco, drugs and weapons, including firearms and accessories, firearm parts or attachments, or ammunition; Content from or about unverified, unapproved or rogue online pharmacies; Offers, attempts or instructions to bypass purchasing laws and regulation; Instructions for creating lethal or toxic substances…" (<https://policy.pinterest.com/en/community-guidelines>)

*Reddit*

“Keep it legal, and avoid posting illegal content or soliciting or facilitating illegal or prohibited transactions." (<https://www.redditinc.com/policies/content-policy>)

“Community content tags are tags that moderators add to their communities to let redditors know what kind of mature content is in that community… Reddit moderators set the content tags for their communities by taking a quick survey about how a community’s posts and discussions currently involve the following mature themes: Amateur advice; Alcohol & tobacco; Drug use; Gambling; Guns & weapons; Nudity; Profanity; Sex & eroticism; Violence” (<https://support.reddithelp.com/hc/en-us/articles/360048185132-What-are-community-content-tags-and-how-do-they-work>)

*TikTok*

“While adults make personal choices about how they engage with alcohol, drugs, and tobacco, we recognize that there are risks connected to trading and using these substances. We do not allow showing or promoting recreational drug use, or the trade of alcohol, tobacco products, and drugs. We also recognize that using these substances can put young people at a heightened risk of harm. We do not allow showing or promoting young people possessing or consuming alcohol, tobacco products, and drugs. Content is age-restricted and ineligible for the For You Feed (FYF) if it shows adults consuming excessive amounts of alcohol or tobacco products.”

“NOT allowed: Showing or promoting young people possessing or consuming alcohol, tobacco products, drugs, or other regulated substances; Showing or promoting adults consuming drugs or other regulated substances for a recreational purpose; Showing the misuse of common household items or over-the-counter products to get intoxicated, such as antihistamines, nutmeg, nitrous oxide canisters, and sniffing glue; Providing instructions on how to make homemade spirits, drugs, or other regulated substances; Facilitating the trade or purchase of alcohol, tobacco products, drugs, or other regulated substances.”

“Allowed: Raising awareness about substance misuse and sharing recovery stories; Advocating for the reform of drug policies and regulations.”

(<https://www.tiktok.com/community-guidelines/en/regulated-commercial-activities>)

*Tumblr*

“Don’t use Tumblr for any kind of exchange of regulated drugs, substances, devices, goods, or weapons. Don't use Tumblr to buy them, sell them, trade them, or to share instructions for manufacturing them." (<https://www.tumblr.com/policy/en/community>)

*X (Twitter)*

“You may not use our service for any unlawful purpose or in furtherance of illegal activities. This includes selling, buying, or facilitating transactions in illegal goods or services, as well as certain types of regulated goods or services." (<https://help.twitter.com/en/rules-and-policies/regulated-goods-services>)

*YouTube*

“[T]he following content isn’t allowed on YouTube: Hard drug use or creation: Hard drug use or creation, selling or facilitating the sale of hard or soft drugs, facilitating the sale of regulated pharmaceuticals without a prescription, or showing how to use steroids in non-educational content. Generally, hard drugs are defined as drugs that can lead to physical addiction, such as cocaine or opioids. Soft drugs include marijuana and salvia.”

“Here are some examples of content that’s not allowed on YouTube… Content instructing how to purchase drugs on the dark web… Including a link to an online pharmacy that does not require prescriptions. Content that promotes a product that contains drugs, nicotine, or a controlled substance. Displays of hard drug use: Non-educational content that shows the injection of intravenous drugs like heroin or huffing/sniffing glue. Making hard drugs: Non-educational content that explains how to make drugs.” (<https://support.google.com/youtube/answer/9229611>)

**Appendix H. Current data access policies, by platform (as of December 1, 2023)**

*Bluelight*

Bluelight.org has a dedicated research portal in alignment with its mission to be a resource for research into drug use and harm reduction. The research portal offers services to researchers and is a starting point for collaboration with the platform. The organization notes that the discussions posted on their site are “cited in the scientific literature published on novel and emerging drugs." Discussions are public and available for research use. However, user demographic data is released in a Bluelight census report, with the most recent being in 2018.

Bluelight contains strict guidelines about data usage:

"If you would like to use Bluelight content as data in your research project, we respectfully wish to remind you that unauthorized reproduction or use of content from Bluelight is explicitly prohibited by our User Agreement. Projects of this nature can be conducted with our permission and ideally in partnership with us. We welcome new ideas about research that uses our data and we encourage you to contact our research team to discuss your ideas further."

"All messages posted become the exclusive property of Bluelight.org. Unauthorized reproduction or use is prohibited. Bluelight partners with both public and private research organizations and we encourage researchers to contact us for more information on our research standards and protocols."

*Drugs-forum*

Drugs-forum.com has no available API. The content was generally available for research purposes, as it was previously archived on Substance Abuse and Mental Health Data Archive.

*Facebook*

Facebook currently operates under an open research & transparency (FORT) policy, which provides curated research datasets (*e.g.* ad targeting transparency, URL sharing, civic engagement data) through a research-specific API. There is an application process to access these data, and access is structured through a remote computing instance where researchers can access and analyze through Jupyterlab. Facebook claims to provide "near real-time data as well as billions of historical data points including raw, anonymized data from public forums on Facebook including Groups, Events & Pages."

*Instagram*

Instagram offers an API for app developers. Instagram shares basic user demographics including self-reported gender, age and broad location with developers, although access for research purposes varies. There are several third party API products for accessing these data, although Instagram warns that "there are a handful of pre-built web scraping packages for Python and R that are specifically designed to collect data from Instagram. Usage of these tools will violate the Terms of Service on the account you use for the scraping and it may be banned from the platform."

*LinkedIn*

LinkedIn offers an API, with different versions for free and premium users. Data includes post content and metadata, user information, learning platform data, and job listings. LinkedIn claims that the API itself allows third parties to retrieve information from LinkedIn without any restrictions.

*Pinterest*

Pinterest has no academic or research API, but does offer an analytics interface. Information shared includes metadata regarding pins, including data abouts apps, products, recipes, and articles tied to each pin. User related information is collected, but is used for internal purposes, with only public information such as username being available in the API. There are several third-party APIs that claim to have access to demographic data such as user age and location, but these are unverified and may violate the Pinterest user agreement.

*Reddit*

Reddit data is limited in the demographic outcomes that it can provide. Reddit provides posts, comments, and user information such as handle name and number of posts. Data was formerly available through Pushshift, a third-party API. However, since Reddit started charging for API use, Pushshift has gone out of operation. Reddit has its own API, but it is rate limited to 100 queries per minute.

*TikTok*

TikTok has a research API available to people who have "demonstrable academic experience and expertise in the research area specified in the application," are employed at a non-profit academic institution, and have a clearly defined research proposal. PhD students need to submit an endorsement letter from an advisor. TikTok shares "all the videos that are: made public by a creator who is aged 18 and over; are posted in the regions of US, Europe and Rest of the World; and do not belong to Canada."

*Tumblr*

Tumblr provides a first-party API with no dedicated research or academic product. Current data from the API includes posts, comments, hashtags as well as usernames and users post data such as volume and number of posts.

*X*

X provides a first-party API, with products that vary in cost and data availability. There is an Academic Research product available to users that are affiliated with an academic institution and have a clearly defined research objective. Data available through both APIs are rapidly changing in the current landscape. Currently, the Academic Research API provides “free access to the full history of public conversation via the full-archive search endpoint, which was previously limited to paid premium or enterprise customers.”

*YouTube*

YouTube has an open-access first-party API as well as a scalable Research API. The research API allows for greater volume of data access but requires an application procedure. It does not allow access to private data but does grant “expanded access to global video metadata across the entire public YouTube.” The data that is available is largely centered around videos, including content, views, and subscriptions to channels. YouTube also offers an audience demographic option that allows content creators to see the age, gender and geography through their analytics portal, although access to these data is limited.

**Appendix I. Supplementary references**

Abouchedid R, Gilks T, Dargan PI, Archer JRH, Wood DM. Assessment of the Availability, Cost, and Motivations for Use over Time of the New Psychoactive Substances-Benzodiazepines Diclazepam, Flubromazepam, and Pyrazolam-in the UK. J Med Toxicol. 2018 Jun;14(2):134–43.

Al-Garadi MA, Yang YC, Guo Y, Kim S, Love JS, Perrone J, et al. Large-Scale Social Media Analysis Reveals Emotions Associated with Nonmedical Prescription Drug Use. Health Data Sci. 2022;2022:9851989.

Al-Rawi A. The convergence of social media and other communication technologies in the promotion of illicit and controlled drugs. J Public Health. 2022 Mar 1;44(1):e153–60.

Alambo A, Padhee S, Banerjee T, Thirunarayan K. COVID-19 and Mental Health/Substance Use Disorders on Reddit: A Longitudinal Study [Preprint]. arXiv; 2020 [cited 2024 Mar 14]. Available from: <https://arxiv.org/abs/2011.10518v1>

Anderson L, Bell HG, Gilbert M, Davidson JE, Winter C, Barratt MJ, et al. Using Social Listening Data to Monitor Misuse and Nonmedical Use of Bupropion: A Content Analysis. JMIR Public Health Surveill. 2017 Feb 1;3(1):e6.

Anwar M, Khoury D, Aldridge AP, Parker SJ, Conway KP. Using Twitter to Surveil the Opioid Epidemic in North Carolina: An Exploratory Study. JMIR Public Health Surveill. 2020 Jun 24;6(2):e17574.

Arillotta D, Guirguis A, Corkery JM, Scherbaum N, Schifano F. COVID-19 Pandemic Impact on Substance Misuse: A Social Media Listening, Mixed Method Analysis. Brain Sci. 2021 Jul 9;11(7):907.

Arshonsky J, Krawczyk N, Bunting AM, Frank D, Friedman SR, Bragg MA. Informal Coping Strategies Among People Who Use Opioids During COVID-19: Thematic Analysis of Reddit Forums. JMIR Form Res. 2022 Mar 3;6(3):e32871.

Atkinson AM, Matthews BR, Nicholls E, Sumnall H. ‘Some days I am a lunatic that thinks I can moderate’: Amalgamating recovery and neo-liberal discourses within accounts of non-drinking among women active in the ‘positive sobriety’ community on Instagram in the UK. Int J Drug Policy. 2023 Feb;112:103937.

Balsamo D, Bajardi P, Panisson A. Firsthand Opiates Abuse on Social Media: Monitoring Geospatial Patterns of Interest Through a Digital Cohort. In: The World Wide Web Conference [Internet]. 2019 [cited 2024 Mar 14]. p. 2572–9. Available from: <http://arxiv.org/abs/1904.00003>

Black JC, Margolin ZR, Olson RA, Dart RC. Online Conversation Monitoring to Understand the Opioid Epidemic: Epidemiological Surveillance Study. JMIR Public Health Surveill. 2020 Jun 29;6(2):e17073.

Blok D, Ambrose L, Ouellette L, Seif E, Riley B, Judge B, et al. Selling poison by the bottle: Availability of dangerous substances found on eBay. Am J Emerg Med. 2020 Apr 1;38(4):846–8.

Boling KS, Habecker P, Kirkpatrick CE, Hample J, Subramanian R, Schlosser A, et al. “Addiction is Not a Choice.” #narcansaveslives: Collective Voice in Harm Reduction on TikTok. Health Commun. 2024 Jun 11;1–11.

Bremer W, Plaisance K, Walker D, Bonn M, Love JS, Perrone J, et al. Barriers to opioid use disorder treatment: A comparison of self-reported information from social media with barriers found in literature. Front Public Health. 2023;11:1141093.

Brynjolfsson E, Collis A. How Should We Measure the Digital Economy? Harvard Business Review [Internet]. 2019 Nov 1 [cited 2024 Mar 14]; Available from: https://hbr.org/2019/11/how-should-we-measure-the-digital-economy

Bunting AM, Krawczyk N, Lippincott T, Gu Y, Arya S, Nagappala S, et al. Trends in Fentanyl Content on Reddit Substance Use Forums, 2013-2021. J Gen Intern Med. 2023 Nov;38(15):3283–7.

Calac AJ, McMann T, Cai M, Li J, Cuomo R, Mackey TK. Exploring substance use disorder discussions in Native American communities: a retrospective Twitter infodemiology study. Harm Reduct J. 2022 Dec 14;19:141.

Catalani V, Arillotta D, Corkery JM, Guirguis A, Vento A, Schifano F. Identifying New/Emerging Psychoactive Substances at the Time of COVID-19; A Web-Based Approach. Front Psychiatry. 2020;11:632405.

Chan B, Lopez A, Sarkar U. The Canary in the Coal Mine Tweets: Social Media Reveals Public Perceptions of Non-Medical Use of Opioids. PloS One. 2015;10(8):e0135072.

Chancellor S, Nitzburg G, Hu A, Zampieri F, De Choudhury M. Discovering Alternative Treatments for Opioid Use Recovery Using Social Media. In: Proceedings of the 2019 CHI Conference on Human Factors in Computing Systems [Internet]. New York, NY, USA: Association for Computing Machinery; 2019 [cited 2024 Mar 14]. p. 1–15. (CHI ’19). Available from: https://dl.acm.org/doi/10.1145/3290605.3300354

Chary M, Genes N, Giraud-Carrier C, Hanson C, Nelson LS, Manini AF. Epidemiology from Tweets: Estimating Misuse of Prescription Opioids in the USA from Social Media. J Med Toxicol. 2017 Dec 1;13(4):278–86.

Chen AT, Johnny S, Conway M. Examining stigma relating to substance use and contextual factors in social media discussions. Drug Alcohol Depend Rep. 2022 Jun;3:100061.

Chen X, Faviez C, Schuck S, Lillo-Le-Louët A, Texier N, Dahamna B, et al. Mining Patients’ Narratives in Social Media for Pharmacovigilance: Adverse Effects and Misuse of Methylphenidate. Front Pharmacol. 2018 May 24;9:541.

Chenworth M, Perrone J, Love JS, Graves R, Hogg-Bremer W, Sarker A. Methadone and suboxone mentions on twitter: thematic and sentiment analysis. Clin Toxicol Phila Pa. 2021 Nov;59(11):982–91.

Cherian R, Westbrook M, Ramo D, Sarkar U. Representations of Codeine Misuse on Instagram: Content Analysis. JMIR Public Health Surveill. 2018 Mar 20;4(1):e22.

Clendennen SL, Loukas A, Vandewater EA, Perry CL, Wilkinson AV. Exposure and engagement with tobacco-related social media and associations with subsequent tobacco use among young adults: A longitudinal analysis. Drug Alcohol Depend. 2020 Aug;213:108072.

Colditz JB, Hsiao LH, Bergman BG, Best DW, Hulsey EG, Sidani JE, et al. Characteristics and engagement among English-language online forums for addiction recovery available in the US. Internet Interv. 2024 Mar;35:100708.

Correia RB, Li L, Rocha LM. Monitoring Potential Drug Interactions and Reactions via Network Analysis of Instagram User Timelines. Pac Symp Biocomput. 2016;21:492–503.

Cuomo R, Purushothaman V, Calac AJ, McMann T, Li Z, Mackey T. Estimating County-Level Overdose Rates Using Opioid-Related Twitter Data: Interdisciplinary Infodemiology Study. JMIR Form Res. 2023 Jan 25;7:e42162.

D’Agostino AR, Optican AR, Sowles SJ, Krauss MJ, Lee KE, Cavazos-Rehg PA. Social networking online to recover from opioid use disorder: A study of community interactions. Drug Alcohol Depend. 2017 Dec 1;181:5–10.

Drug Enforcement Administration. Social Media Drug Trafficking Threat [Internet]. 2022. Available from: https://www.dea.gov/sites/default/files/2022-03/20220208-DEA_Social%20Media%20Drug%20Trafficking%20Threat%20Overview.pdf

Egan KG, Moreno MA. Alcohol references on undergraduate males’ Facebook profiles. Am J Mens Health. 2011 Sep;5(5):413–20.

Eichstaedt JC, Schwartz HA, Kern ML, Park G, Labarthe DR, Merchant RM, et al. Psychological Language on Twitter Predicts County-Level Heart Disease Mortality. Psychol Sci. 2015 Feb;26(2):159–69.

El-Bassel N, Hochstatter KR, Slavin MN, Yang C, Zhang Y, Muresan S. Harnessing the Power of Social Media to Understand the Impact of COVID-19 on People Who Use Drugs During Lockdown and Social Distancing. J Addict Med. 2022 Apr 1;16(2):e123–32.

Elphinston RA, Scotti Requena S, Angus D, De Andrade D, Freeman CR, Day MA. The Promotion of Policy Changes Restricting Access to Codeine Medicines on Twitter: What do National Pain Organizations Say? J Pain. 2020 Jul;21(7–8):881–91.

ElSherief M, Sumner S, Krishnasamy V, Jones C, Law R, Kacha-Ochana A, et al. Identification of Myths and Misinformation About Treatment for Opioid Use Disorder on Social Media: Infodemiology Study. JMIR Form Res. 2024;8:e44726.

ElSherief M, Sumner SA, Jones CM, Law RK, Kacha-Ochana A, Shieber L, et al. Characterizing and Identifying the Prevalence of Web-Based Misinformation Relating to Medication for Opioid Use Disorder: Machine Learning Approach. J Med Internet Res. 2021 Dec 22;23(12):e30753.

Flores L, Young SD. Regional Variation in Discussion of Opioids on Social Media: A Qualitative Study. J Addict Dis. 2021;39(3):316–21.

Fodeh SJ, Al-Garadi M, Elsankary O, Perrone J, Becker W, Sarker A. Utilizing a multi-class classification approach to detect therapeutic and recreational misuse of opioids on Twitter. Comput Biol Med. 2021 Feb;129:104132.

Frank D, Krawczyk N, Arshonsky J, Bragg MA, Friedman SR, Bunting AM. COVID-19-Related Changes to Drug-Selling Networks and Their Effects on People Who Use Illicit Opioids. J Stud Alcohol Drugs. 2023 Mar;84(2):222–9.

Fung ICH, Blankenship EB, Ahweyevu JO, Cooper LK, Duke CH, Carswell SL, et al. Public Health Implications of Image-Based Social Media: A Systematic Review of Instagram, Pinterest, Tumblr, and Flickr. Perm J. 2020;24:18.307.

Garg S, Taylor J, El Sherief M, Kasson E, Aledavood T, Riordan R, et al. Detecting risk level in individuals misusing fentanyl utilizing posts from an online community on Reddit. Internet Interv. 2021 Dec;26:100467.

Giorgi S, Yaden DB, Eichstaedt JC, Ungar LH, Schwartz HA, Kwarteng A, et al. Predicting U.S. county opioid poisoning mortality from multi-modal social media and psychological self-report data. Sci Rep. 2023 Jun 3;13(1):9027.

Glowacki EM, Glowacki JB, Wilcox GB. A Text-Mining Analysis of the Public’s Reactions to the Opioid Crisis. Subst Abuse. 2018 Apr 1;39(2):129–33.

Glowacki EM, Wilcox GB, Glowacki JB. Identifying #Addiction Concerns on Twitter during the COVID-19 Pandemic: A Text Mining Analysis. Subst Abuse. 2021 Jan;42(1):39–46.

Goyer C, Castillon G, Moride Y. Implementation of Interventions and Policies on Opioids and Awareness of Opioid-Related Harms in Canada: A Multistage Mixed Methods Descriptive Study. Int J Environ Res Public Health. 2022 Apr 22;19(9):5122.

Graves RL, Tufts C, Meisel ZF, Polsky D, Ungar L, Merchant RM. Opioid Discussion in the Twittersphere. Subst Use Misuse. 2018 Nov 10;53(13):2132–9.

Grund JPC, Latypov A, Harris M. Breaking worse: The emergence of krokodil and excessive injuries among people who inject drugs in Eurasia. Int J Drug Policy. 2013 Jul;24(4):265–74.

Guidry J, Jin Y, Haddad L, Zhang Y, Smith J. How Health Risks Are Pinpointed (or Not) on Social Media: The Portrayal of Waterpipe Smoking on Pinterest. Health Commun. 2016;31(6):659–67.

Haber I, Pergolizzi J, LeQuang JA. Poppy Seed Tea: A Short Review and Case Study. Pain Ther. 2019 Jun;8(1):151–5.

Hanson CL, Cannon B, Burton S, Giraud-Carrier C. An Exploration of Social Circles and Prescription Drug Abuse Through Twitter. J Med Internet Res. 2013 Sep 6;15(9):e189.

Hassanpour S, Tomita N, DeLise T, Crosier B, Marsch LA. Identifying substance use risk based on deep neural networks and Instagram social media data. Neuropsychopharmacology. 2019 Feb;44(3):487–94.

Haupt MR, Cuomo R, Li J, Nali M, Mackey TK. The influence of social media affordances on drug dealer posting behavior across multiple social networking sites (SNS). Comput Hum Behav Rep. 2022 Dec 1;8:100235.

Holborn T, Schifano F, Deluca P. No prescription? No problem: A qualitative study investigating self-medication with novel psychoactive substances (NPS). Int J Drug Policy. 2023 Aug;118:104109.

Ittefaq M, Zain A, Bokhari H. Opioids in Satirical News Shows: Exploring Topics, Sentiments, and Engagement in Last Week Tonight on YouTube. J Health Commun. 2023 Jan 2;28(1):53–63.

Jancey J, Leaver T, Wolf K, Freeman B, Chai K, Bialous S, et al. Promotion of E-Cigarettes on TikTok and Regulatory Considerations. Int J Environ Res Public Health. 2023 May 9;20(10):5761.

Jha D, Singh R. Analysis of associations between emotions and activities of drug users and their addiction recovery tendencies from social media posts using structural equation modeling. BMC Bioinformatics. 2020 Dec 30;21(Suppl 18):554.

Jones DM, Guy MC, Soule E, Sakuma KLK, Pokhrel P, Orloff M, et al. Characterization of Electronic Cigarette Warning Statements Portrayed in YouTube Videos. Nicotine Tob Res. 2021 Aug 4;23(8):1358–66.

Katselou M, Papoutsis I, Nikolaou P, Spiliopoulou C, Athanaselis S. 5‐(2‐aminopropyl)indole: A new player in the drama of ‘legal highs’ alerts the community. Drug Alcohol Rev. 2015 Jan;34(1):51–7.

Kazemi DM, Borsari B, Levine MJ, Dooley B. Systematic review of surveillance by social media platforms for illicit drug use. J Public Health. 2017 Dec 1;39(4):763–76.

Kepner W, Meacham MC, Nobles AL. Types and Sources of Stigma on Opioid Use Treatment and Recovery Communities on Reddit. Subst Use Misuse. 2022;57(10):1511–22.

Kilgo DK, Midberry J. Social Media News Production, Emotional Facebook Reactions, and the Politicization of Drug Addiction. Health Commun. 2022 Mar;37(3):375–83.

Kim E. Etsy blocks sales of drugs and human remains. CNN Business [Internet]. 2012 Aug 10; Available from: https://money.cnn.com/2012/08/10/technology/etsy-bans-drugs/index.html

Krauss MJ, Sowles SJ, Mylvaganam S, Zewdie K, Bierut LJ, Cavazos-Rehg PA. Displays of dabbing marijuana extracts on YouTube. Drug Alcohol Depend. 2015 Oct;155:45–51.

Krawczyk N, Bunting AM, Frank D, Arshonsky J, Gu Y, Friedman SR, et al. “How will I get my next week’s script?” Reactions of Reddit opioid forum users to changes in treatment access in the early months of the coronavirus pandemic. Int J Drug Policy. 2021 Jun;92:103140.

Krawczyk N, Miller M, Gu EY, Irvine N, Ramirez E, Santaella‐Tenorio J, et al. Self‐reported experiences and perspectives on using psychedelics to manage opioid use among participants of two Reddit communities. Addiction. 2025 Jan 16;add.16767.

Laestadius L, Wang Y. Youth access to JUUL online: eBay sales of JUUL prior to and following FDA action. Tob Control. 2019 Nov;28(6):617–22.

Laestadius LI, Guidry JPD, Greskoviak R, Adams J. Making “Weedish Fish”: An Exploratory Analysis of Cannabis Recipes on Pinterest. Subst Use Misuse. 2019;54(13):2191–7.

Lavertu A, Hamamsy T, Altman RB. Monitoring the opioid epidemic via social media discussions [Preprint]. 2021 [cited 2024 Mar 14]. p. 2021.04.01.21254815. Available from: https://www.medrxiv.org/content/10.1101/2021.04.01.21254815v1

Lee AS, Hart JL, Sears CG, Walker KL, Siu A, Smith C. A picture is worth a thousand words: Electronic cigarette content on Instagram and Pinterest. Tob Prev Cessat. 2017 Jul 3;3:119.

Lee JY, Lee YS, Kim DH, Lee HS, Yang BR, Kim MG. The Use of Social Media in Detecting Drug Safety–Related New Black Box Warnings, Labeling Changes, or Withdrawals: Scoping Review. JMIR Public Health Surveill. 2021 Jun 28;7(6):e30137.

Lindeman M, Katainen A, Svensson J, Kauppila E, Hellman M. Compliance with regulations and codes of conduct at social media accounts of Swedish alcohol brands. Drug Alcohol Rev. 2019 May;38(4):386–90.

Liu T, Giorgi S, Yadeta K, Schwartz HA, Ungar LH, Curtis B. Linguistic predictors from Facebook postings of substance use disorder treatment retention versus discontinuation. Am J Drug Alcohol Abuse. 2022 Sep 3;48(5):573–85.

Magge A, Tutubalina E, Miftahutdinov Z, Alimova I, Dirkson A, Verberne S, et al. DeepADEMiner: a deep learning pharmacovigilance pipeline for extraction and normalization of adverse drug event mentions on Twitter. J Am Med Inform Assoc. 2021 Sep 18;28(10):2184–92.

Matero M, Giorgi S, Curtis B, Ungar LH, Schwartz HA. Opioid death projections with AI-based forecasts using social media language. Npj Digit Med. 2023 Mar 8;6(1):1–11.

Meacham MC, Nobles AL, Tompkins DA, Thrul J. “I got a bunch of weed to help me through the withdrawals”: Naturalistic cannabis use reported in online opioid and opioid recovery community discussion forums. PloS One. 2022;17(2):e0263583.

Merten JW, Gordon BT, King JL, Pappas C. Cannabidiol (CBD): Perspectives from Pinterest. Subst Use Misuse. 2020;55(13):2213–20.

Moyle L, Childs A, Coomber R, Barratt MJ. #Drugsforsale: An exploration of the use of social media and encrypted messaging apps to supply and access drugs. Int J Drug Policy. 2019 Jan 1;63:101–10.

Naserianhanzaei E, Koschate-Reis M. Effects of Substance Use, Recovery, and Non-Drug-Related Online Community Participation on the Risk of a Use Episode During Remission From Opioid Use Disorder: Longitudinal Observational Study. J Med Internet Res. 2022 Aug 22;24(8):e36555.

Nasralah T, El-Gayar O, Wang Y. Social Media Text Mining Framework for Drug Abuse: Development and Validation Study With an Opioid Crisis Case Analysis. J Med Internet Res. 2022 Aug 13;22(8):e18350.

Nguyen T, Larsen ME, O’Dea B, Phung D, Venkatesh S, Christensen H. Estimation of the prevalence of adverse drug reactions from social media. Int J Med Inf. 2017 Jun;102:130–7.

Nikfarjam A, Sarker A, O’Connor K, Ginn R, Gonzalez G. Pharmacovigilance from social media: mining adverse drug reaction mentions using sequence labeling with word embedding cluster features. J Am Med Inform Assoc. 2015 Mar 1;22(3):671–81.

Nobles AL, Johnson DC, Leas EC, Goodman-Meza D, Zúñiga ML, Ziedonis D, et al. Characterizing Self-Reports of Self-Identified Patient Experiences with Methadone Maintenance Treatment on an Online Community during COVID-19. Subst Use Misuse. 2021;56(14):2134–40.

O’Brien EK, Hoffman L, Navarro MA, Ganz O. Social media use by leading US e-cigarette, cigarette, smokeless tobacco, cigar and hookah brands. Tob Control. 2020 Mar 26;tobaccocontrol-2019-055406.

O’Kelly B, Holmes P, Cheng A, Lee JD, Tofighi B. Dissemination of health content through social networks: YouTube and opioid use disorders. J Subst Use Addict Treat. 2024 Oct;165:209475.

Paul MJ, Chisolm MS, Johnson MW, Vandrey RG, Dredze M. Assessing the Validity of Online Drug Forums as a Source for Estimating Demographic and Temporal Trends in Drug Use. J Addict Med. 2016 Oct;10(5):324.

Preiss A, Baumgartner P, Edlund MJ, Bobashev GV. Using Named Entity Recognition to Identify Substances Used in the Self-medication of Opioid Withdrawal: Natural Language Processing Study of Reddit Data. JMIR Form Res. 2022 Mar 30;6(3):e33919.

Price R. Etsy is awash with illicit products it claims to ban, from ivory to dangerous weapons and mass-produced goods. Business Insider [Internet]. 2021 Apr 30; Available from: https://www.businessinsider.com/etsy-sells-ivory-weapons-poisonous-plants-mass-produced-products-2021-4

Primack BA, Colditz JB, Rosen EB, Giles LM, Jackson KM, Kraemer KL. Portrayal of Alcohol Brands Popular Among Underage Youth on YouTube: A Content Analysis. J Stud Alcohol Drugs. 2017 Sep;78(5):654–64.

Purushothaman V, Li J, Mackey TK. Detecting Suicide and Self-Harm Discussions Among Opioid Substance Users on Instagram Using Machine Learning. Front Psychiatry. 2021;12:551296.

Raj S, Ghosh A, Pandiyan S, Chauhan D, Goel S. Analysis of YouTube content on substance use disorder treatment and recovery. Int J Soc Psychiatry. 2023 Dec;69(8):2097–109.

Rajendra-Nicolucci C, Zuckerman E. Top 100: The most popular social media platforms and what they can teach us [Internet]. 2021. Available from: <https://knightcolumbia.org/blog/top-100-the-most-popular-social-media-platforms-and-what-they-can-teach-us>

Rajesh K, Wilcox G, Ring D, Mackert M. Reactions to the opioid epidemic: A text-mining analysis of tweets. J Addict Dis. 2021;39(2):183–8.

Ramachandran S, Brown L, Ring D. Tones and themes in Reddits posts discussing the opioid epidemic. J Addict Dis. 2022;40(4):552–8.

Rhumorbarbe D, Morelato M, Staehli L, Roux C, Jaquet-Chiffelle DO, Rossy Q, et al. Monitoring new psychoactive substances: Exploring the contribution of an online discussion forum. Int J Drug Policy. 2019 Nov 1;73:273–80.

Russell AM, Bergman BG, Colditz JB, Kelly JF, Milaham PJ, Massey PM. Using TikTok in recovery from substance use disorder. Drug Alcohol Depend. 2021 Dec;229:109147.

Rutherford BN, Sun T, Johnson B, Co S, Lim TL, Lim CCW, et al. Getting high for likes: Exploring cannabis-related content on TikTok. Drug Alcohol Rev. 2022 Jul;41(5):1119–25.

Sarker A, Al-Garadi MA, Ge Y, Nataraj N, Jones CM, Sumner SA. Signals of increasing co-use of stimulants and opioids from online drug forum data. Harm Reduct J. 2022 May 25;19(1):51.

Sarker A, Ginn R, Nikfarjam A, O’Connor K, Smith K, Jayaraman S, et al. Utilizing Social Media Data for Pharmacovigilance: A Review. J Biomed Inform. 2015 Apr;54:202–12.

Sarker A, Gonzalez-Hernandez G, Ruan Y, Perrone J. Machine Learning and Natural Language Processing for Geolocation-Centric Monitoring and Characterization of Opioid-Related Social Media Chatter. JAMA Netw Open. 2019 Nov 6;2(11):e1914672.

Sarker A, Nataraj N, Siu W, Li S, Jones CM, Sumner SA. Concerns among people who use opioids during the COVID-19 pandemic: a natural language processing analysis of social media posts. Subst Abuse Treat Prev Policy. 2022 Mar 5;17(1):16.

Shah N, Li J, Mackey TK. An unsupervised machine learning approach for the detection and characterization of illicit drug-dealing comments and interactions on Instagram. Subst Abuse. 2022;43(1):273–7.

Sloane R, Osanlou O, Lewis D, Bollegala D, Maskell S, Pirmohamed M. Social media and pharmacovigilance: A review of the opportunities and challenges. Br J Clin Pharmacol. 2015;80(4):910–20.

Soussan C, Kjellgren A. The users of Novel Psychoactive Substances: Online survey about their characteristics, attitudes and motivations. Int J Drug Policy. 2016 Jun 1;32:77–84.

Squires NA, Soyemi E, Yee LM, Birch EM, Badreldin N. Content Quality of YouTube Videos About Pain Management After Cesarean Birth: Content Analysis. JMIR Infodemiology. 2023 Jun 23;3:e40802.

Stokes DC, Purtle J, Meisel ZF, Agarwal AK. State Legislators’ Divergent Social Media Response to the Opioid Epidemic from 2014 to 2019: Longitudinal Topic Modeling Analysis. J Gen Intern Med. 2021 Nov;36(11):3373–82.

Sumner SA, Bowen D, Holland K, Zwald ML, Vivolo-Kantor A, Guy GP Jr, et al. Estimating Weekly National Opioid Overdose Deaths in Near Real Time Using Multiple Proxy Data Sources. JAMA Netw Open. 2022 Jul 21;5(7):e2223033.

Sun T, Lim CCW, Chung J, Cheng B, Davidson L, Tisdale C, et al. Vaping on TikTok: a systematic thematic analysis. Tob Control. 2023 Mar;32(2):251–4.

Tacheva Z, Ivanov A. Exploring the Association Between the “Big Five” Personality Traits and Fatal Opioid Overdose: County-Level Empirical Analysis. JMIR Ment Health. 2021 Mar 8;8(3):e24939.

Thanki D, Frederick BJ. Social media and drug markets. In Luxembourg: Publications Office of the European Union; 2016 [cited 2024 Mar 14]. p. 115–23. Available from: http://www.emcdda.europa.eu/system/files/publications/2155/TDXD16001ENN_FINAL.pdf

Tibebu S, Chang VC, Drouin CA, Thompson W, Do MT. At-a-glance - What can social media tell us about the opioid crisis in Canada? Health Promot Chronic Dis Prev Can Res Policy Pract. 2018 Jun;38(6):263–7.

Tofighi B, Perna M, Desai A, Grov C, Lee J. Craigslist as a source for heroin: a report of two cases. J Subst Use. 2016;21(5):543–6.

Vosburg SK, Dailey-Govoni T, Beaumont J, Butler SF, Green JL. Characterizing the Experience of Tapentadol Nonmedical Use: Mixed Methods Study. JMIR Form Res. 2022 Jun 10;6(6):e16996.

Wanchoo K, Abrams M, Merchant RM, Ungar L, Guntuku SC. Reddit language indicates changes associated with diet, physical activity, substance use, and smoking during COVID-19. Doran N, editor. PLOS ONE. 2023 Feb 3;18(2):e0280337.

Whelan J, Noller GE, Ward RD. Rolling through TikTok: An analysis of 3,4-methylenedioxymethamphetamine-related content. Drug Alcohol Rev. 2024 Jan;43(1):36–44.

Wightman RS, Perrone J, Erowid F, Erowid E, Meisel ZF, Nelson LS. Comparative Analysis of Opioid Queries on Erowid.org: An Opportunity to Advance Harm Reduction. Subst Use Misuse. 2017 Aug 24;52(10):1315–9.

[Wikipedia] List of social platforms with at least 100 million active users. In: Wikipedia [Internet]. 2024 [cited 2024 Mar 14]. Available from: <https://en.wikipedia.org/w/index.php?title=List_of_social_platforms_with_at_least_100_million_active_users&oldid=1213672777>

Williams RS. Underage internet alcohol sales on eBay. Addiction. 2013;108(7):1346–8.

Wu J, Origgi JM, Ranker LR, Bhatnagar A, Robertson RM, Xuan Z, et al. Compliance With the US Food and Drug Administration’s Guidelines for Health Warning Labels and Engagement in Little Cigar and Cigarillo Content: Computer Vision Analysis of Instagram Posts. JMIR Infodemiology. 2023 Mar 14;3:e41969.

Wu J, Trifiro BM, Ranker LR, Origgi JM, Benjamin EJ, Robertson RM, et al. Health Warnings on Instagram Advertisements for Synthetic Nicotine E-Cigarettes and Engagement. JAMA Netw Open. 2024 Sep 13;7(9):e2434434.

Yang CC, Yang H, Jiang L. Postmarketing Drug Safety Surveillance Using Publicly Available Health-Consumer-Contributed Content in Social Media. ACM Trans Manag Inf Syst. 2014 Apr 1;5(1):2:1-2:21

Yang X, Luo J. Tracking Illicit Drug Dealing and Abuse on Instagram Using Multimodal Analysis. ACM Trans Intell Syst Technol. 2017 Feb 24;8(4):58:1-58:15.

Yang YC, Al-Garadi MA, Love JS, Cooper HLF, Perrone J, Sarker A. Can accurate demographic information about people who use prescription medications nonmedically be derived from Twitter? Proc Natl Acad Sci. 2023 Feb 21;120(8):e2207391120.

Yang Z, Nguyen L, Jin F. Predicting Opioid Relapse Using Social Media Data [Preprint]. arXiv; 2018 [cited 2024 Mar 14]. Available from: http://arxiv.org/abs/1811.12169

Yao H, Rashidian S, Dong X, Duanmu H, Rosenthal RN, Wang F. Detection of Suicidality Among Opioid Users on Reddit: Machine Learning-Based Approach. J Med Internet Res. 2020 Nov 27;22(11):e15293.

Yeh J, Villani J, Haikalis M, Rios M, Pielech M. #Opioids: A Mixed Methods Examination of Top Opioid-Related Content on TikTok. J Stud Alcohol Drugs. 2024 Dec 27;jsad.23-00418.

Zaprutko T, Kopciuch D, Paczkowska A, Sprawka J, Cynar J, Pogodzińska M, et al. Facebook as a source of access to medicines. PloS One. 2022;17(10):e0275272.

Zhao F, Skums P, Zelikovsky A, Sevigny EL, Swahn MH, Strasser SM, et al. Computational Approaches to Detect Illicit Drug Ads and Find Vendor Communities Within Social Media Platforms. IEEE/ACM Trans Comput Biol Bioinform. 2022;19(1):180–91.

**Equation A. Per-platform Formal Normalized Ratio**

$$\text{Formal Normalized Ratio}\left( \text{platform} \right)= \frac{\sum_{\text{formal opioid terms}} \# \text{term hits on platform}}{\sum_{\text{household terms}} \text{\# term hits on platform}}*100,000$$

**Equation B. Per-platform Informal Normalized Ratio**

$$\text{Informal Normalized Ratio}\left( \text{platform} \right)= \frac{\sum_{\text{informal opioid terms}} \# \text{term hits on platform}}{\sum_{\text{household terms}} \text{\# term hits on platform}}*100,000$$

**Equation C. Per-platform Algospeak Normalized Ratio**

$$\text{Algospeak Normalized Ratio}\left( \text{platform} \right)= \frac{\sum_{\text{algospeak opioid terms}} \# \text{term hits on platform}}{\sum_{\text{household terms}} \text{\# term hits on platform}}*100,000$$
