# Supplementary figures and images for: Which social media platforms facilitate monitoring the opioid crisis?
Source: PLOS Digit Health. 2025 Apr 28;4(4):e0000842. doi: 10.1371/journal.pdig.0000842 (PMC12036940; doi:10.1371/journal.pdig.0000842)

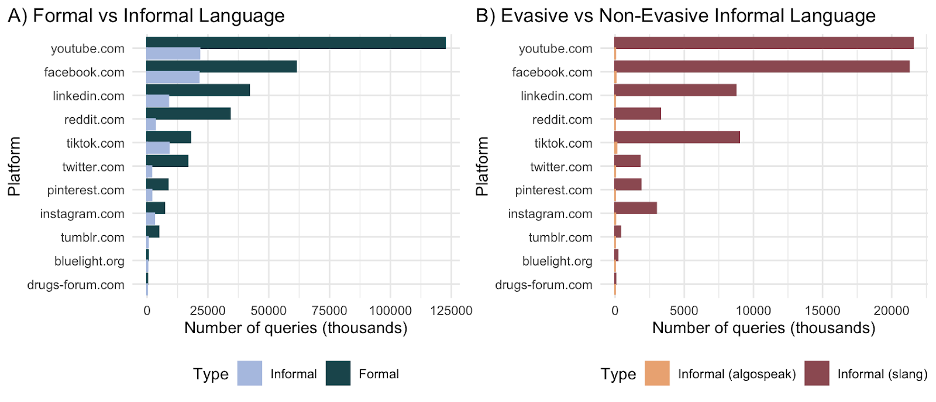

Supplement: S1 Fig — Listed by social media platform. A) Comparison between formal and informal/algospeak language. B) Comparison between informal and algospeak language. (PNG) [file pdig.0000842.s002.png]
